# Tau as a potential therapeutic target for ischemic stroke

**DOI:** 10.18632/aging.102547

**Published:** 2019-12-16

**Authors:** Xin Chen, Hua Jiang

**Affiliations:** 1 Department of Geriatrics, Shanghai East Hospital, Tongji University School of Medicine, Shanghai, China

**Keywords:** tau, ischemic stroke, phosphorylation, therapy

## Abstract

Tau is a protein mainly expressed in adult human brain. It plays important roles both in neurodegenerative diseases and stroke. Stroke is an important cause of adult death and disability, ischemic stroke almost account for 80% in all cases. Abundant studies have proven that the increase of dysfunctional tau may act as a vital factor in pathological changes after ischemic stroke. However, the relationship between tau and ischemic stroke remains ununified. Based on present studies, we firstly introduced the structure and biological function of tau protein. Secondly, we summarized the potential regulatory mechanisms of tau protein in the process of ischemic stroke. Thirdly, we discussed about the findings in therapeutic researches of ischemic stroke. This review may be helpful in implementing new therapies for ischemic stroke and may be beneficial for the clinical and experimental studies.

## INTRODUCTION

Nowadays, stroke has become the leading cause of adult disability and the second most prominent cause of death worldwide, only after coronary heart disease. Ischemic stroke almost accounts for 80% in all stroke cases [1]. Over the past decades, the established therapeutic option for ischemic stroke patients is still limited to recanalization of occlusive vessels with the clot-breaking agent tissue plasminogen activator (t-PA). However, due to the serious tissue damage which may occur during the subsequent reperfusion (such as bleeding) and the limited therapeutic time window (within 4.5h post stroke), more than 90% of ischemic stroke patients are unavailable to intravenous t-PA therapy [2]. Although numerous potential pathophysiologic mechanisms and targets for ischemic stroke have been found in recent years, they are rarely translated into feasible medical practice [3].

**Figure 1:**
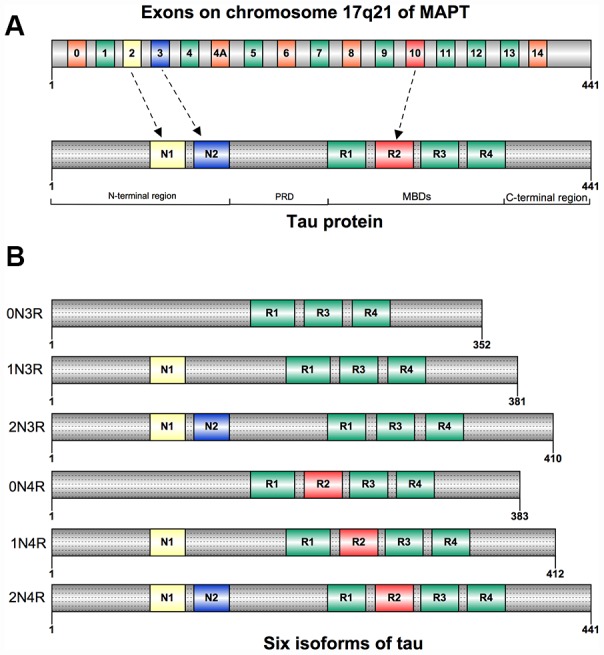
(**A**) Structure of human tau protein; Tau has an N-terminao projection region, a proline-rich domain(PRD), a microtubule-binding domain(MBD), and a C-terminal region. (**B**) Six isoforms of human tau. They differ by the inclusion of exon 2(NI), exon 3(N2), and exonlO(RI-R4).

Tau is a protein mainly expressed in the brain, it has six isoforms produced by alternative mRNA splicing of microtubule-associated protein tau (MAPT) gene which comprises 16 exons on chromosome 17q21 [4] (Figure 1A). The primary physiological function of tau protein is to stabilize microtubule networks within neurons, whereas the hyperphosphorylated condition will significantly reduce its biological activity [5]. Although previous studies mainly focused on the mechanisms of tau protein in neurodegenerative diseases [6, 7], some studies have also demonstrated that increased tau immunoreactivity after brain ischemia-reperfusion injury can be observed in neuronal cells [8, 9]. Recently, several novel functions of tau protein have been revealed [10, 11]. Whereas the association between tau protein and ischemic stroke has not been well discussed. In this review, we aim to update the knowledge about the genomic and proteomic changes in tau protein following ischemia/reperfusion injury and the connection between tau protein and ischemic stroke.

## Structure and biological functions of tau

Tau was first isolated and named in 1975 for its ability to induce tubule formation [12], and was mostly segregated into neuronal axons [13]. Tau can be also detected in oligodendrocytes and neuronal somatodendritic compartments [14]. Besides the nervous system, tau was also found to be expressed in many other tissues: heart, lung, kidney, and testis, but less abundant [15]. Tau is composed of four regions: an N-terminal projection region, a proline-rich domain (PRD), a microtubule-binding domain (MBD), and a C-terminal region [16] (Figure 1A). Six isoforms of tau have been found in human adult brain, they are expressed by alternative splicing around the N-terminal projection region and MBD. The gene expression of these isoforms differs both in N-terminal exons (0N, 1N, or 2N) and the number of microtubule binding repeat sequences (3R or 4R). The 4R tau has four microtubule binding repeat sequences due to the inclusion of exon 10 when compared with 3R tau [4, 17] (Figure 1B).

**Figure 2:**
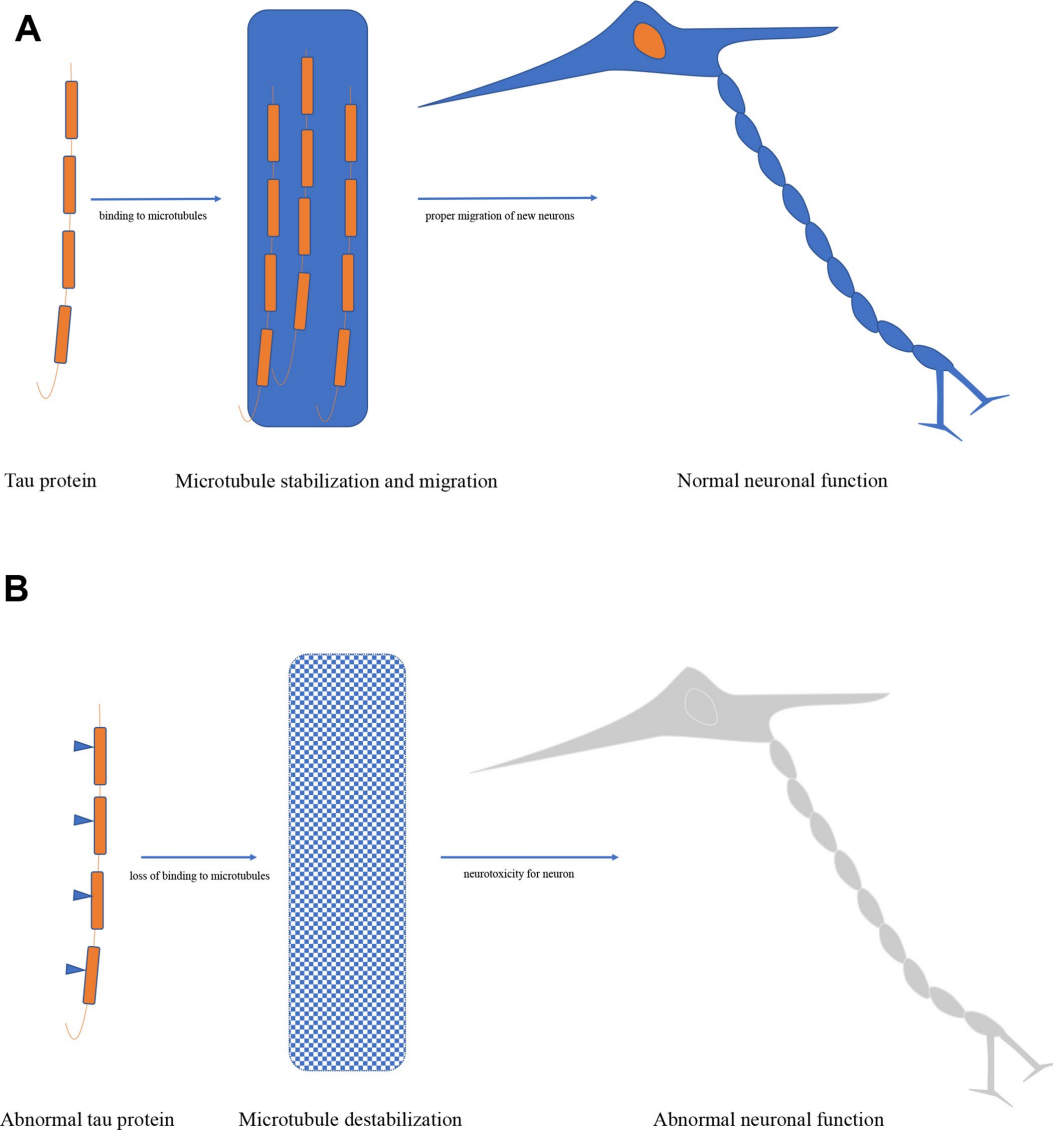
(**A**) Biological function of tau protein. (**B**) Pathological role of tau protein.

The mainly physiological tau function in the cell is regulating microtubule structure and dynamics by binding to microtubules, it has been also proven in cell-free conditions [12]. Furthermore, the dynamic microtubule network provided by tau is key to the proper migration of new neurons, and severe reduction of adult neurogenesis was found in tau knockout mice [18]. Tau also plays an important role in controlling the balance of microtubule-dependent axonal transport through the differential sensitivity of motor proteins in neurons [19]. Additionally, it has been approved that tau is essential in the protection of neuronal genomic DNA and RNA integrity under hyperthermia condition both in primary neuronal cultures and *in vivo* in adult mice [20]. Besides, absence or reduction of tau expression has been reported to have protective efforts against memory deficits, excitotoxicity, amyloid induced toxicity, and epilepsy in animal experiments [21, 22] (Figure 2).

## Potential mechanisms of tau in ischemic stroke

Tau functions are regulated by a complex array of post-translational modifications, such as phosphorylation, glycation, acetylation, isomerization, nitration, sumoylation, O-GlcNAcylation, and truncation [16, 23], suggesting that tau plays diverse roles in physiology and pathology. Dysfunctional tau is one of the neurotoxic proteins, accumulated in neurons and cerebrovascular after ischemia, furthermore, it is closely related to a range of pathological changes of ischemic stroke [24, 25]. According to previous studies, the kinds of dysfunctional tau differ in different ischemic models, such as neurofibrillary tangle formation [26–28], hyperphosphorylation [29–34], dephosphorylation [8, 35–39], and re-phosphorylation [8, 40] (Table 1). The hyperphosphorylated state is the particularly pathological condition of tau in brain ischemia. It decreases the affinity of tau for the microtubules by disrupting the binding balance [5, 30–34, 41]. In this part, we will summarize the potential regulatory mechanisms of tau in ischemic stroke.

**Table 1:** Patterns of Tau Phosphorylation in Brain after Ischemic Stroke

**References**	**Human/Animal**	**Models/Subjects**	**Ischemic time**	**Analyzed tissue**	**State of tau protein**	**Tau phospho-sites**	**Effects of tau**
Bi M 2017 [11]	Mice	Focal cerebral ischemia model	90min/ 30min	The cortex in the ischemic area	Tau	N	Reduce tau protein-dependent excitotoxicity in tau–/– mice
Basurto IG 2018 [117]	Mice	Focal cerebral ischemia model	1 hour	The ischemic core	Hyperphosphorylation	Ser262/356	Hyperphosphorylation involving asparagine endopeptidase
Khan S 2018 [27]	Mice	Global cerebral ischemia model	10,15,18min	The hippocampus and the cortex	Paired helical filament tau protein increase	Ps396/404	Lead to neuronal death
Liao G 2009 [118]	Mice	Right common carotid artery was occluded and hypoxia was maintained	40 min	The ischemic core	A marked decrease in tau phosphorylation	P301L	Extracellular glutamate accumulation
Tuo QZ 2017 [10]	Mice/ Rats	Focal cerebral ischemia model	Mice:60min Rats:90min	The lesioned hemisphere	Tau	N	Dysfunctional or absent tau protein contributes to iron-mediated neurotoxicity
Dewar D 1995 [36]	Rats	Focal cerebral ischemia model	2-6hours	The cortex in the ischemic area	Dephosphorylated and/or degraded	Tau 1	Breakdown of the cytoskeleton in ischemic region of the neuron
Geddes JW 1994 [37]	Rats	Complete cerebral ischemia model	20 min	The hippocampal formation	Dephosphorylated	Tau 1	Compromises the ability of the neuron to remove Elevated intracellular Ca2+
Shackelford DA,1998 [39]	Rats	Complete cerebral ischemia model	5-15min	The hippocampus, neocortex and striatum	Dephosphorylated	Ps396/404	Possibly contributing to disruption of axonal transport
Wen Y 2004 [31]	Rats	Focal cerebral ischemia model	1 hour	The cortex in the ischemic area	Hyperphosphorylation	PT181, pS202, pT205, pT212, pS214, pT231, pS262, pS396, pS404, and pS422	Destabilize neuronal cytoskeleton, and may contribute to the Apoptotic process
Wen Y 2004 [33]	Rats	Focal cerebral ischemia model	1 hour	The cortex in the ischemic area	Hyperphosphorylation	MC1 and TG3 (phospho-tau 231/ 235); phosphorylated tau epitopes: CP13 (phospho- tau 202/205), CP3 (phospho-tau 214), PHF-1 (phospho-tau 396/ 404), and CP9 (phospho-tau 231)	Involved in the progression of Neuropathology in AD
Kovalska M 2018 [34]	Rats	Global cerebral ischemia model	15min	The cortex in the ischemic area	Hyperphosphorylation	Ser202, Thr205	Degeneration of cortical neurons, alterations in number and morphology of tissue astrocytes and dysregulation of Oxidative balance
Fujii H 2017 [30]	Rats	Focal cerebral ischemia model	90 mins	The ischemic core	Hyperphosphorylation	Asp421-truncated tau	Influence microtubule stability and Subsequently disturb axonal transport, resulting in the formation of axonal varicosities and other axonal abnormalities
Wen Y 2007 [29]	Rats	Focal cerebral ischemia model	1 hour	The cortex in the ischemic area	Hyperphosphorylation and neurofibrillary tangle (NFT) like conformations	P-396/404	Involved in the progression of neuropathology in AD
Majd S 2016 [38]	Rats	Global cerebral ischemia model	8 mins	Parietal cortical and subcortical hippocampus homogenates	Phosphorylation/ dephosphorylation	Ser(396) and Ser(262), Ser(202) /Thr(205) (AT8)	Dephosphorylation of AMPK followed the same pattern as tau dephosphorylation during ischemia or reperfusion
Whitehead SN,2005 [28]	Rats	Subcortical Lacunar infarcts by striatal endothelin injections	N	Hippocampus	Neurofibrillary tangles and senile plaques to form	Tau 2	Mediating neurotoxic and neuroinflammatory
Morioka M 2006 [32]	Gerbils	Global forebrain ischemia model	5 mins	Hippocampal region	Hyperphosphorylation	Serine 199/202	Induced by MAP kinase, CDK5, and GSK3, and contributes to ischemic neuronal injury
Gordon KW 2007 [8]	Gerbils	Global forebrain ischemia model	5 mins	The cortex in the ischemic area	Hyperphosphorylation	Tau 1	May caused by oxidative stress
Mailliot C 2000 [40]	Dogs	Cardiac arrest -induced global cerebral ischemia	10mins	The ischemic core	Dephosphorylation, differential and re-phosphorylation	Ser262/356	Monitor neuronal integrity after brain ischemia
Burkhart KK 1998 [35]	Rats/Human	Complete cerebral ischemia model Neocortical brain slices	5mins/ 30mins	The cortex in the ischemic area	Dephosphorylation and an apparent recovery in phosphorylated tau	Tau 1	Dephosphorylated tau may enhance Microtubule stability
Uchihara T 2004 [127]	Human	Ischemic stroke	N	The cortex in the ischemic area	Hyperphosphorylation	Ser101	Microglia tau protein passes independent of phosphorylation modification
Kato T 1988 [26]	Human	Ischemic stroke	N	The cortex in the ischemic area	Neurofibrillary tangle formation	Tau 1	These cases may represent an initial stage of senile changes

### Tau and oxidative stress

Oxidative stress is a pathological condition which constitutes the mechanisms of many disease including ischemic stroke. It has been proven in animal experiments that the hyperphosphorylation of tau can be resulted from oxidative stress through different kinds of oxidant, like intracerebroventricular streptozotocin (ICV-STZ) [42, 43], streptozotocin [44] and 1,2- diacetylbenzene (DAB) [45]. On the other hand, hyperphosphorylation of tau can be reduced by antioxidants, such as EUK 207 [46], EUK 189 [47] and exendin-4 (Ex-4) [42]. There is no unified opinion on the underlying mechanisms between oxidative stress and hyperphosphorylation of tau. Many studies have found that polyunsaturated lipids, thiobarbituric acid reactive substances (TBARS), and 4-hydroxynonenal (4-HNE) produced by peroxidation of intracellular lipids are notably increased, which may contribute to hyperphosphorylation of tau [42, 43]. More recently, tau hyperphosphorylation is proven to be directly stimulated by ROS, which is produced by DAB via the phosphorylation of activated glycogen synthase kinase-3β (GSK-3β) [45]. Moreover, high concentration of hyperphosphorylated tau has been shown to stimulate the production of ROS [48]. Therefore, oxidative stress and tau hyperphosphorylation may be two key elements of a vicious circle after ischemic stroke.

### Tau and apoptosis

Apoptosis is a dynamically programmed process of cell death, acting an essential actor in the neuronal damage after ischemic stroke [49]. Tau hyperproteolysis/ proteolysis and apoptosis are considered to be two independent pathological events after neuron damage, most researchers did not demonstrate the underlying relationship between them [50, 51]. However, one recent study has proven that the accumulation of CDK5-regulated tau phosphorylation might trigger neuronal apoptosis through impairing endoplasmic reticulum-associated degradation [52]. Researchers also found that tau phosphorylation could be inhibited by knocking down CDK5 (an upstream regulatory factor of tau), which could protect neurons by mitigating endoplasmic reticulum stress from apoptosis [52].

### Tau and autophagy

Autophagy is subtyped into constitutive macro-autophagy which plays a major role in maintaining the appropriate levels of functional tau in neurons [53–55]. Autophagy has been indicated to be an important pathophysiological process in both hemorrhagic stroke and ischemic stroke [56, 57]. Previous studies have demonstrated that the decrease in tau is directly correlated with the increase in specific autophagy markers (such as LC3B-II) in the 3xTg-AD mouse model after transient hypoperfusion, indicating that autophagy may be a pathway of lowering dysfunctional tau level after hypoperfusion [58]. Another study has detected a significant decrease in the level of LC3B protein and a reduction in infarct volume in ischemic P301L-Tau mice [59]. The researchers considered it might be possible that the autophagy-mediated degradation is influenced by mutated tau with the increase levels of protein aggregates [59]. Furthermore, it has been demonstrated that regulators of autophagy can mediate tau expression in neurons overexpressing human mutant P301L-Tau [60]. In human tauopathies, p62 is the regulative protein of selective autophagy, and its immunoreactivity co-localizes with tau inclusions [61]. In mice and cells, autophagy activation can promote the clearance of assembled tau [62] and reduce the aggregation of seeded tau [63]. Many studies consider tau phosphorylation a consequence of seeded aggregation [64]. P62 and nuclear dot protein 52 (NDP52) are both autophagy cargo receptors, playing vital role in protecting against seeded tau aggregation in cells [60, 65]. So it is possible that autophagy, rather than the proteasome, restricts the aggregation of seeded tau [60].

### Tau and excitotoxicity

Excitotoxicity has been identified as one of the molecular mechanisms of ischemic stroke in many studies [66–68]. Many studies suggest that tau phosphorylation can be prevented by inhibition of calcium influx [69]. The increased activity of calcium-dependent kinases or altered glutamate homeostasis can enhance tau phosphorylation [70, 71], meaning the glutamate-induced excitotoxicity can increase dysfunctional tau expression. On the other hand, several other studies find that tau also plays a critical role in eliciting excitotoxicity [72–77]. There is an increase in KCL-evoked glutamate release and a decrease in glutamate clearance in TauP301L mice [74]. The molecular mechanisms underlying tau-induced excitotoxicity remain elucidated. A latter study demonstrates tau facilitates excitotoxicity with a mechanism that does not directly involve facilitation of calcium influx through kainic acid (KA) receptors [78]. However, another study suggests that the reduction of the pY18-tau formation or level can depress excitotoxicity by diminishing N-methyl-D-aspartic acid (NMDA) receptor-dependent calcium influx [79, 80]. Altogether, excitotoxicity and tau phosphorylation lead to a vicious circle in promoting cell death in ischemic brain.

### Tau protein and inflammation

Inflammation of neural tissue, also called neuroinflammation, is considered the main cause of mortality in ischemia/reperfusion stroke [81]. Some previous studies have suggested that dysfunctional tau is closely related to inflammatory cascade. The inflammatory messengers can significantly affect the structure and function of tau [82–84]. The misfolded tau can represent a trigger for inflammatory cascade [82–84]. The exact roles of inflammatory processes on tau pathology or dysfunctional tau on inflammation still remain unequivocal. Some researchers generally consider inflammation an exacerbating factor [83], but another study also shows that acute inflammation may decrease the oligomeric tau levels by improving the ability of microglia [85]. The first direct evidence for the role of inflammation on tau pathology was demonstrated in a vitro study in 2003. This study showed that the inflammatory mediator, interleukin-1β (IL-1β), could promote tau phosphorylation via activating p38-mitogen-activated protein kinases (MAPK) [86]. In the same year, this role was confirmed in a vivo study with the 3xTg model [87]. The latter studies also showed that a series of bacterial or viral immune stressors and tumor necrosis factors could trigger an increase in tau phosphorylation [88–90]. So reducing tau levels or inhibiting inflammatory pathways could serve as a way to resist tauopathies [91]. In 2009, Kovac et al. found a novel toxic gain of function of misfolded tau, truncated tau. Truncated tau could induce significant decrease of trans-endothelial electrical resistance and increase of endothelial permeability of BBB. Further, researchers also found that truncated tau showed cytotoxic effects on astrocyte-microglia culture manifested by increased extracellular adenylate kinase levels. Blood-brain barrier damage induced by truncated tau was mediated through pro-inflammatory cytokine TNF-α and chemokine MCP-1 [23]. It is noteworthy that pro-inflammatory cytokine interferon-γ (IFNγ) has been reported to have opposing effects on the phosphorylation and dephosphorylation of tau [92]. The macrophages and microglia play a vital role in neuroinflammation. Tau oligomers can only be phagocytosed by both macrophages and microglia under physiological condition [85]. Microglial internalization has been indicated to be effective to both soluble and aggregated human tau [93]. Overall, suppressing the inflammation in neural tissue may prove paradoxically effective in the development of tau pathology. Further studies are required to elucidate the molecular mechanism.

### Tau protein and angiogenesis

Vascular endothelium refers to cells that line the entire circulatory system. It has a close relationship with thrombosis and thrombolysis. Dysfunctional endothelium plays a key role in the pathology of stroke by increasing the atherosclerotic plaques size and vulnerability [94]. Previous studies have suggested that endothelial cells can be damaged by tauopathy, such as hyperphosphorylation and insolubility, via decreasing microtubule assembly [95–98]. Measurement of cerebral perfusion in different studies indicate that tau pathology is related to reduced blood flow [99, 100]. Truncated tau has been proven to play an important role in regulating permeability of BBB by decreasing transendothelial electrical resistance (TEER) and increasing mannitol permeability [23]. In aged tau-overexpressing mice, tau pathological changes can impact the brain endothelial cell biological function by influencing the integrity of the brain’s microvasculature [101]. Furthermore, researchers in this study also find the accumulation of pathological tau is related to the expression of hypoxia-and/or angiogenesis-related genes, such as Serpine1, Vegfa, Plau and Hmox1 [101]. However, the precise cellular signals of these changes and the specific interactions between tau and endothelial cells still remain further elucidated. Therefore, tau pathology may play an important role in the process of BBB disruption and neurogenesis by regulating activities of endothelial cells after ischemic stroke.

### Tau protein and mitochondrial dysfunction

Neuronal cells are particularly sensitive to energy deficiencies. The function of mitochondria is to maintain the energy supply for cells. Mitochondrial dysfunction is one of the pivotal pathological processes in brain ischemia and reperfusion. Mitochondrial dysfunction then causes neurons necrosis, autophagy and apoptosis [102]. Disruption of mitochondrial dynamics (the balance between fission and fusion) is the core factor in mitochondrial dysfunction. Previous studies showed that dynamin-related protein 1 (DRP1), a kind of mitochondrial fission proteins, could interact with phosphorylated tau, leading to mitochondrial dysfunction [103, 104]. Meanwhile, reducing Drp1 levels could protect against mitochondrial dysfunction induced by hyperphosphorylated tau [105]. Additionally, a significant association between tau accumulation and mitochondrial translocation deficits was found both in the mouse models and human brains [106]. The abnormal mitochondrial trafficking can be improved through reducing soluble tau levels [106]. In cell and animal studies, overexpressed tau can both destroy physiological function and distribution of mitochondria, which may cause ATP exhausting, oxidative stress and synaptic dysfunction [107–109]. In the mechanism studies, glycogen synthase kinase 3 (GSK3), axonal protein phosphatase 1 (PP1), and phosphorylated tau trapped kinesin motor protein complex JIP1 were considered to be involved in the pathological interaction [110, 111]. It is interesting to notice that tau phosphorylation can also be aggravated by ROS mimicking mitochondrial oxidative stress in neuronal cells [112]. Altogether, tau pathology can destroy the mitochondrial dynamics and function, while the dysfunctional mitochondria may indicate tau phosphorylation and aggregation.

### Tau protein and neurovascular unit damage

The abnormal neuron-to-neuron connections and dysfunctional interactions among the different components in the neurovascular unit (NVU) might be the main reasons for functional deficits after ischemic stroke [113]. A study found that ischemia could induce neurovascular alterations, glial changes, and the loss of tight junctions in NVU, leading to the BBB breakdown [9]. By immunofluorescence assay, they also confirmed the Aβdeposits and dysfunctional tau existed with glial reactions and morphologically altered endothelia [9]. Therefore, tau may play an important role in the process of NVU damage after ischemic stroke. In the future, a focus on all components and investigation of intercellular signaling and signaling between cells and extracellular matrix is essential to clarify all the facts about ischemic stroke.

In summary, we have discussed the potential mechanisms of tau in ischemic stroke, including oxidative stress, apoptosis, autophagy, excitotoxicity, inflammation, endothelium and angiogenesis, and mitochondrial dysfunction. In addition, we also discussed the role of tau in NVU damage. Tau may stand at the intersection of multiple regulatory mechanisms for major pathological changes in ischemic stroke.

## Therapeutic researches

From the above, it is clear that intervention in tau-mediated pathological changes could be considered as a clinically beneficial strategy in ischemic stroke. No such therapy related to tau-regulation have yet achieved regulatory approval for clinical application and further evidence is still required. However, there has been many studies achieved encouraging progress. They found the reduction in tau activities and levels might have clinical benefits in stroke treatment. In this part, we will mainly discuss the findings in animal and clinical studies.

### Tau in animal studies

In animal studies, tau hyperphosphorylation was found in rats after ischemic damage, and this was considered the consequence of the activation/ inactivation of a variety of phosphatases and kinases [31]. In addition, tau hyperphosphorylation could be caused by hypoxia-dependent mechanism in vascular dysfunction models, such as the ischemic model [114]. Focal mild hypothermia is considered a protective factor on ischemia/reperfusion damage. It can significantly reduce the neurotoxicity by influencing the level of tau in rats [115]. In 2017, two important preclinical studies involving transient middle cerebral artery occlusion (MCAO) mouse models suggested novel roles for tau in acute ischemic injury, indicating that agents targeting tau and related proteins have the potential to reduce the severity of acute brain damage following stroke [10, 11]. Peng Lei and colleagues found no elevated brain iron or reperfusion injury in young (3-month-old) tau–/– mice after MCAO. While this protection was lost in older (12-month-old) tau–/– mice: the brain iron accumulated rapidly. However, the protective effects of tau knockout could be revived through normalizing the iron elevation during the reperfusion phase. They suggested the interaction between tau and iron might be pleiotropic modulators of ischemic stroke [10]. Ittner and co-workers found no up-regulation of the immediate-early genes Arc, Fos and Junb in tau–/– mice after MCAO damage. But the levels of their mRNA were higher in tau+/+ brains [11]. They also demonstrated several signaling pathways were differently activated between tau–/– mice and tau+/+ mice, mitogen-activated protein kinase (MAPK) pathway was the most notable one. Then, the inhibitor of excitotoxic RAS/ERK signaling in tau–/– mice, SynGAP1, was found significantly increased at the post-synapse in the investigation of the MAPK pathway. This study demonstrated that tau and SynGAP1 might be potential targets for acute ischemic stroke [11]. Some other studies also show that inhibitor 2 of protein phosphatase 2A (I_2_PP2A) can produce hyperphosphorylation of tau through inhibition of PP2A [116] in MCAO mouse model [117]. Increased level of glutamate transporter 1 in transgenic mouse model can reduce ischemic brain damage through reducing the accumulation of extracellular glutamate and the activation of subsequent calpain and caspase [118].

### Tau in clinical studies

In clinical studies, an increase of the total tau level was found in human cerebrospinal fluid(CSF) after brain injury, including ischemic stroke [119, 120]. Meanwhile, tau was found measurable in serum within 6 h after ischemic symptom onset [121]. The concentration might peak after 3–5 days [121], or later [122]. Moreover, there was no statistical correlation between tau serum levels and the severity of clinical deficit or disability as assessed by the Barthel index (BI). But the serum levels of tau were correlated with infarct volumes (from 7ml to 48ml) and functional outcomes after 90 days [121]. The results were consistent with other studies which indicated that the absence of tau in serum during the acute phase (<24h) of ischemia could predict good clinical outcomes in 90 days after stroke [123]. Patients with detected tau in serum got more severe neurological deficits and worse functional outcomes when compared with patients without tau [124]. However, other researchers discovered tau protein levels were correlated with the scored neurological deficits (BI) from 48 h onward. They also found the serum tau levels had no significant correlation with stroke etiology as represented by the TOAST-criteria [125]. A recent prospective study proved that tau levels were closely related to not only stroke severity as assessed by NIHSS, but also long-term outcomes both in plasma and CSF [126]. Notably, the study of autopsied brains from patients with cerebral infarction found that an increase of tau immunoreactivity and deposition of tau in ischemic area, but tau deposits were not organized into fibrils or more solid inclusions indicating that tau epitope was secondary to ischemic damage [127, 128]. Nevertheless, tau can be detected in the serum of approximately 40% of stroke patients [121, 122]. Present studies have not proven why tau could not be detected in the blood of all stroke patients. Some researchers think tau may occur in blood due to the disruption of BBB. Some factors like MMP9 may play a key role in the release of tau into circulation [122].

At present, researchers have found several methods to reduce the tau aggregation or tau levels. Methylene blue was considered a tau aggregation blocker. It could induce autophagy and attenuate tauopathy in vitro and in vivo [129], to block tau aggregation in C. elegans [130], although its exact mechanism of action is still not understood. The AMPK-related kinase Nuak1 has been identified as a regulator of tau levels. Inhibition of Nuak1 in fruit flies suppressed tau-dependent neurodegeneration [131]. Moreover, several approaches have targeted the putative enzymes that are responsible for tau changes, such as ERK inhibitor [132], JNK inhibitor [133], GSK3β [134]. However, these methods have not been used clinically for ischemic stroke. Further studies need to explore the application of tau-based therapeutic strategies, especially in acute phase of ischemic stroke.

## CONCLUSIONS AND PERSPECTIVES

This review is committed to describe the pathological roles of tau following cerebral ischemia. Tau is a protein that plays a vital role not only in microtubule assembly and stabilization, but also in pathophysiology of ischemic stroke. Initially, we provided a general aspects of tau protein, including descriptions of its structure, physiological functions and pathological functions. Then, we introduce different pathological states of tau protein under ischemic condition. The pathological changes (such as oxidative stress, autophagy, excitotoxicity, inflammation, endothelium and angiogenesis, and mitochondrial dysfunction) of tau protein determine its potential regulatory mechanisms in ischemic stroke. Phosphorylation is the main pathological change of tau in ischemic stroke. Therefore, controlling tau phosphorylation may induce more protective effects under ischemic stimuli. As some experimental results are from mouse model with FTDP-17 mutations, there might be differences between mouse model with FTDP-17 mutations and those with ischemic injury in pathogenetic mechanisms leading to degeneration. Some studies proved that the regional redistribution of tau from the neuropil to neuronal perikarya in their stroke model was thought to share similarity with that occurring in Alzheimer's disease [30]. But the results of molecular changes in FTDP-17 mutations mouse might different in mouse with stroke. Therefore, more researches still need to explore molecular mechanisms in mouse with ischemic injury. Lastly, we discuss about the therapeutic researches on the treatment of stroke with tau protein. The animal studies indicate a role for tau protein in acute ischemic brain damage, suggesting that agents targeting tau and related proteins have the great potential to reduce the severity of brain damage following acute ischemic stroke. The clinical studies show that the level of serum/plasma or CSF tau is related to the stroke severity of clinical deficit and long-term outcomes. The underlying mechanisms of pathological tau-induced side effects during and after ischemia/reperfusion process are complex. There are insufficient clinical studies focused on link between tau protein and ischemic stroke. However, we still believe that revealing the molecular mechanisms of tau in cerebral ischemia and regulating the tau phosphorylation may be conductive to developing a potential novel target for the ischemic stroke therapy.
